# Relation between fentanyl dose and patient state index during spinal anesthesia for elective cesarean section

**DOI:** 10.1186/s40981-016-0056-3

**Published:** 2016-10-19

**Authors:** Hiroki Iwata, Hiroaki Sakai, Souichiro Mimuro, Nako Uozaki, Hiromitsu Yamaguchi, Kenji Takahashi, Yoshito Shiraishi

**Affiliations:** 1 Department of Anesthesiology, Fujieda Municipal General Hospital, Surugadai 4-1-11, Fujieda city, Shizuoka 426-8677 Japan; 2 Department of Anesthesiology and Intensive Care, Hamamatsu University School of Medicine, Handayama 1-20-1, Higashi-ku, Hamamatsu city, Shizuoka 431-3192 Japan

## Abstract

**Background:**

In spinal anesthesia for cesarean section, the addition of fentanyl to the local anesthetic has been reported to improve perioperative analgesia. However, there is only limited knowledge on sedative effects of the added fentanyl. We examined whether the patient state index® (PSI) can detect and present the light sedated level with patients undergoing cesarean section.

**Findings:**

We measured respiratory rate (RR), SpO2, and PSI values. Between child delivery and the completion of the operation, the proportions of time with the PSI values <90 and 80 were calculated. RR <8 breaths/min or SpO2 <95 % was defined as respiratory depression. Respiratory depression was not observed in any patient. At fentanyl doses of 10, 15, and 20 μg, the proportions of time with the PSI <90 were 14.5 ± 20.8, 49.4 ± 35.3, and 71.1 ± 22.9 %, respectively (*P* < 0.01). There were significant differences between 10 and 15 μg (*P* < 0.05), and 10 and 20 μg (*P* < 0.01). Similarly, the proportions of time with the PSI values <80 were 0.5 ± 1.8, 21.1 ± 24.1, and 31.8 ± 32.2 %, respectively (*P* < 0.05). There was a significant difference between 10 and 20 μg (*P* < 0.05).

**Conclusions:**

The PSI values decreased in a dose-dependent manner with increasing dose of fentanyl, but no respiratory depression was observed. The PSI values decreased to less than 90, when fentanyl was administered more than 15 μg. Furthermore, the PSI values decreased to less than 80, when fentanyl was administered 20 μg.

## Findings

### Introduction

In spinal anesthesia for cesarean section, the addition of fentanyl to the local anesthetic has been reported to reduce nausea and discomfort during surgery, prolong the duration of analgesia, and reduce the required amount of the local anesthetic [1–7]. However, there is only limited knowledge on the influences of the added fentanyl on sedative effects. We examined whether patient state index® (PSI) can detect and present changes in the light sedated level. In this preliminary study, we evaluated the relationship between the dose of the added fentanyl and the PSI with patients in spinal anesthesia undergoing cesarean section.

### Subjects and methods

This was a prospective study performed with the approval of the Ethical Committee of Fujieda Municipal General Hospital. The subjects consisted of 32 patients undergoing elective cesarean section under combined spinal-epidural anesthesia. At the time of preoperative consultation, after an explanation of the purpose of this study had been given to each patient, consent to this study was obtained. Patients with a history of neurological disorders and those using oral antipsychotics or antiepileptic drugs were excluded from the subjects. In addition, patients in whom the dermatomal block level after spinal anesthesia was below the fourth thoracic level (Th4) or analgesics were required during the operation were excluded from the subjects.

The patients were divided into three groups according to the fentanyl dose (10, 15, or 20 μg) for addition. No premedication was given. After the application of an electrocardiograph, blood pressure monitor, and pulse oximeter, each patient was placed in the left lateral decubitus position. An epidural catheter was placed at the Th12/L1 level, and 1 % lidocaine (3 mL) was administered as the test dose. No additional dose was administered during the operation. Subsequently, using a 25G Quincke needle, hyperbaric bupivacaine (2.2 mL) mixed with the above doses of fentanyl was administered at the L3/4 interspace into the spinal subarachnoid space. After the administration, the patient was immediately placed in the supine position. Oxygen (5 L) was administered using a mask, and measurements of the PSI initiated using a SedLine® monitor (Masimo Corp., Irvine, CA) applied to the patient’s forehead were started.

For decreases in the blood pressure during the operation, phenylephrine was intravenously administered whenever necessary. The dermatomal block level after spinal anesthesia was confirmed by evaluating the loss of cold sensation with alcohol. When nausea and vomiting developed, metoclopramide (10 mg) was intravenously administered. When an analgesic was necessary, 1 % lidocaine (5 mL) was administered using the epidural catheter. After the completion of the operation, continuous administration of a mixture composed of fentanyl, bupivacaine, and physiological saline was initiated using the epidural catheter.

Following the drug administration into the subarachnoid space, the PSI values, respiratory rate, SpO_2_, child’s Apgar score, umbilical vein pH, and the presence or absence of nausea during the operation were recorded. Between child delivery and the completion of the operation, the proportions of time with the PSI values less than 90 and 80 were calculated. To avoid influences on the PSI, speaking to the subjects was minimized after child delivery. Results are expressed as the mean value ± standard deviation or median value (range). For statistical analysis, one-way analysis of variance (ANOVA) was used, and Scheffe’s test was used for intergroup comparisons. *P* < 0.05 was regarded as significant.

### Results

Thirty patients were included in three groups (10 patients each). Two patients were excluded because of a headache during the operation. No significant difference was observed in the height, body weight, age, gestational age, or surgical time among the three groups (Table 1). The block level before the procedure was Th4 or above in all patients, without differences among the three groups. No patient required a sedative during the operation. There was also no difference in the child’s Apgar score, umbilical vein pH, or the number of patients with nausea among the three groups. No patient showed SpO_2_ <95 % or respiratory rate <8/min (Table 2).

**Table 1:** Patients’ characteristics

	Fentanyl 10 μg	Fentanyl 15 μg	Fentanyl 20 μg	*P* value
Height (cm)	156.5 ± 5.9	157.7 ± 5.8	157.5 ± 6.3	NS
Weight (kg)	59.5 ± 7.1	56.3 ± 7.9	66.7 ± 15.9	NS
Age (year)	29.8 ± 4.5	32.6 ± 4.5	34.2 ± 6	NS
Gestational age (week)	37.8 ± 0.9	38.1 ± 0.7	38.1 ± 1.2	NS
Surgical time (min)	53.1 ± 15.1	60.6 ± 12.3	55 ± 14.8	NS

Values are presented as mean ± SD

*NS* not significantly different

**Table 2:** Spinal block characteristics, analgesic data, and side effects

	Fentanyl 10 μg	Fentanyl 15 μg	Fentanyl 20 μg	*P* value
Block height	Th4 (Th2–Th4)	Th3 (Th2–Th4)	Th4 (Th2–Th4)	NS
Apgar 1 min	8 (7–9)	8	8	NS
Apgar 5 min	10 (9–10)	9	9 (9–10)	NS
Umbilical vein pH	7.34 ± 0.01	7.33 ± 0.03	7.32 ± 0.02	NS
Nausea	0/10 (0 %)	3/10 (30 %)	2/10 (20 %)	NS
Minimum respiratory rate	12 (10–17)	12.5 (8–16)	11 (8–19)	NS

Values are presented as median (range), mean ± SD, or *n* (%)

*NS* not significantly different

**Fig. 1:**
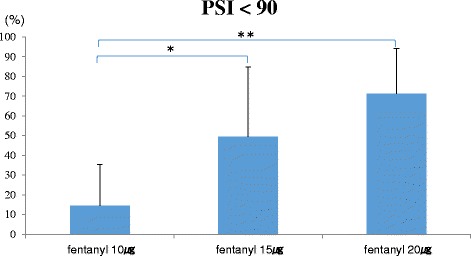
The proportions of time with the PSI values <90. There were significant differences between fentanyl doses of 10 and 15 μg, and fentanyl doses of 10 and 20 μg

**Fig. 2:**
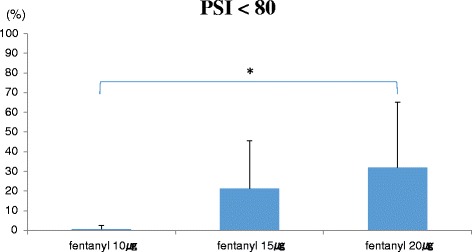
The proportions of time with the PSI values <80. There were significant differences between fentanyl doses of 10 and 20 μg

At fentanyl doses of 10, 15, and 20 μg, the proportions of time with the PSI values <90 were 14.5 ± 20.8, 49.4 ± 35.3, and 71.1 ± 22.9 %, respectively (*P* < 0.01). There were significant differences between 10 and 15 μg (*P* < 0.05), and 10 and 20 μg (*P* < 0.01) (Fig. 1). Similarly, the proportions of time with the PSI values <80 were 0.5 ± 1.8, 21.1 ± 24.1, and 31.8 ± 32.2 %, respectively (*P* < 0.05). There was a significant difference between 10 and 20 μg (*P* < 0.05) (Fig. 2).

### Discussion

The results of the present study demonstrate that PSI values decrease in a dose-dependent manner with increasing dose of fentanyl and respiratory depression was not observed even with an increase in the fentanyl dose to 20 μg.

Spinal anesthesia has been reported to have sedative effects irrespective of the presence or absence of fentanyl addition [6–8]. However, its exact mechanism is unclear, although there are some theories. One theory is that local anesthetics act on the posterior horn of the spinal cord. They interrupt visceral afferent input and decrease stimulation to the reticular activating system which causes antianxiety effects. Another theory is that an increase in the local anesthetic concentration in the central nervous system produces sedative effects [6–9].

There is no established real-time monitor of the depth of sedation. Pollock et al. [6] and Marucci et al. [7] evaluated the sedative effects of spinal anesthesia using the bispectral index® (BIS) and Observer’s Assessment of Alertness/Sedation (OAA/S) scale (Table 3) in 16 healthy volunteers and 23 patients undergoing cesarean section, respectively. Both of them observed a significant decrease in the OAA/S scale score from 5 to 4 but no changes in the BIS value, suggesting that the BIS is not appropriate as a sedation monitor. Johansen and Ouchi surveyed the influences of the OAA/S scale assessment method on the BIS in 10 patients in the ICU. They observed a significant increase in the BIS immediately after assessment when the OAA/S scale score was 2–4, suggesting that the BIS tended to be affected by the assessment stimulation during mild sedation [10] and that the absence of a correlation between the BIS value and OAA/S scale score is due to limitations in the assessment method.

**Table 3:** Observer’s Assessment of Alertness/Sedation scale

Score level	Responsiveness
5	Responds readily to name spoken in a normal tone
4	Lethargic response to name spoken in a normal tone
3	Responds only after name is called loudly and/or repeatedly, or lash reflex is present
2	Loss of lash reflex or has a positive response to train-of-four (TOF) stimulation
1	No purposeful response to TOF stimulation

PSI is a clinically validated measure of the effect of anesthesia. It is a processed parameter of a fourchannel electroencephalograph monitor. Its difference from the bispectral index monitor is that PSI is a processed parameter of a four-channel electroencephalograph monitor instead of two channels in the bispectral index monitor. Concerning the validity of the PSI as a sedation monitor in awake or lightly sedated patients, Drover and Ortega [11] surveyed the relationship between the PSI value and OAA/S score by deepening anesthesia with 0.1 increments in the MAC of an inhaled anesthetic in healthy volunteers. There was a positive correlation between the OAA/S score and PSI value. The PSI value changed even during mild sedation (OAA/S, 5–4) and was about 80 when the OAA/S score was 4. Kurup et al. [8] evaluated the sedative effects using the PSI and OAA/S in 20 patients undergoing urological or orthopedic surgery under spinal anesthesia. They observed a significant decrease in the PSI value from 99 to 78 when the OAA/S score decreased from 5 to 4 and suggested the usefulness of the PSI for assessing the sedative effects of spinal anesthesia. These studies support the validity of the use of the PSI for the evaluation of light sedation in our study.

In this study, the PSI values decrease in a dose-dependent manner with increasing dose of fentanyl. Fentanyl administration at a dose of 20 μg increased the proportions of time with the PSI value less than 80. We did not evaluate the level of consciousness using an objective or subjective score. But, almost all patients seemed calm during surgery after baby birth and replied clearly to our call after the end of the surgery. To our knowledge, there have been no studies which measured the sedated state during the spinal anesthesia for cesarean section by using the PSI value. Marucci et al. [7] studied the sedative effects of spinal anesthesia for cesarean section comparing two groups receiving a local anesthetic with or without the addition of fentanyl (15 μg) by using the OAA/S scale. However, in their study, the fentanyl dose was constant.

In this study, none of the subjects showed maternal respiratory depression. Hunt et al. increased the fentanyl dose added to the local anesthetic stepwise to 50 μg in spinal anesthesia for cesarean delivery but did not observe respiratory depression (respiratory rate, 10 times/min) [2]. Similar results were obtained in the present study. Comparison of the minimum respiratory rate among the three groups showed no significant differences, suggesting negligible influences of fentanyl at a dose of ≤20 μg.

### Conclusion

When fentanyl (10–20 μg) was added to the local anesthetic in spinal anesthesia for cesarean section, the PSI value decreased, but no respiratory depression was observed. In particular, the PSI values decreased to less than 90, when fentanyl was administered more than 15 μg. Furthermore, the PSI values decreased to less than 80, when fentanyl was administered 20 μg. These results indicate that the PSI could be a monitor to detect and present the small change of conscious level caused by the added fentanyl into the local anesthetic in spinal anesthesia for cesarean section.
